# Supplementary figures and images for: CustOmics: A versatile deep-learning based strategy for multi-omics integration
Source: PLoS Comput Biol. 2023 Mar 6;19(3):e1010921. doi: 10.1371/journal.pcbi.1010921 (PMC10019780; doi:10.1371/journal.pcbi.1010921)

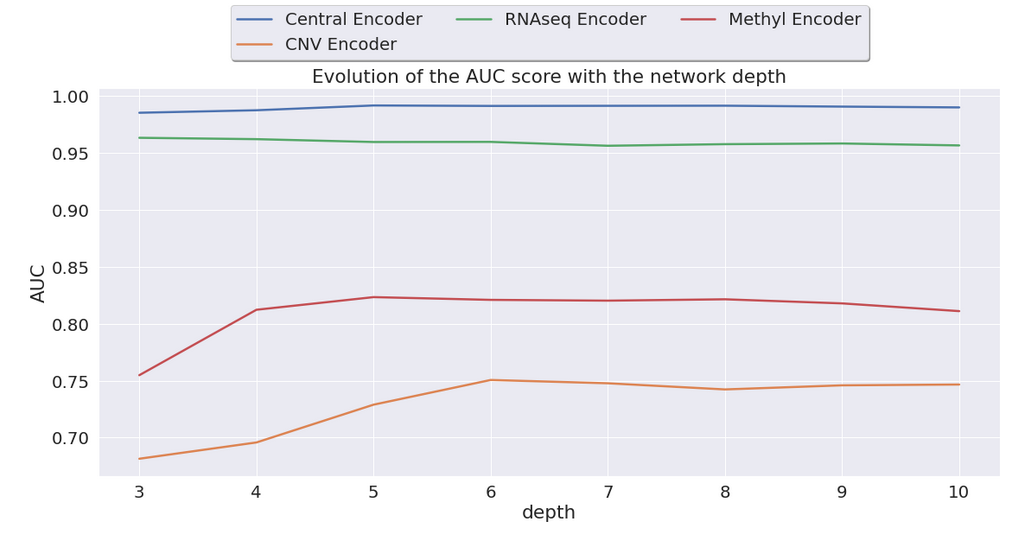

Supplement: S1 Fig — We first assess the evolution of the performance on the tumor classification task for each source using the intermediate autoencoders, then we evaluate the effect of the depth on the central encoder using the best results for the intermediate autoencoders for each source. We see that RNAseq data does not need as many layers as CNV and methylation data, suggesting that its convergence may be simpler as it holds most of the signal for tumor-type prediction. (PNG) [file pcbi.1010921.s006.png]

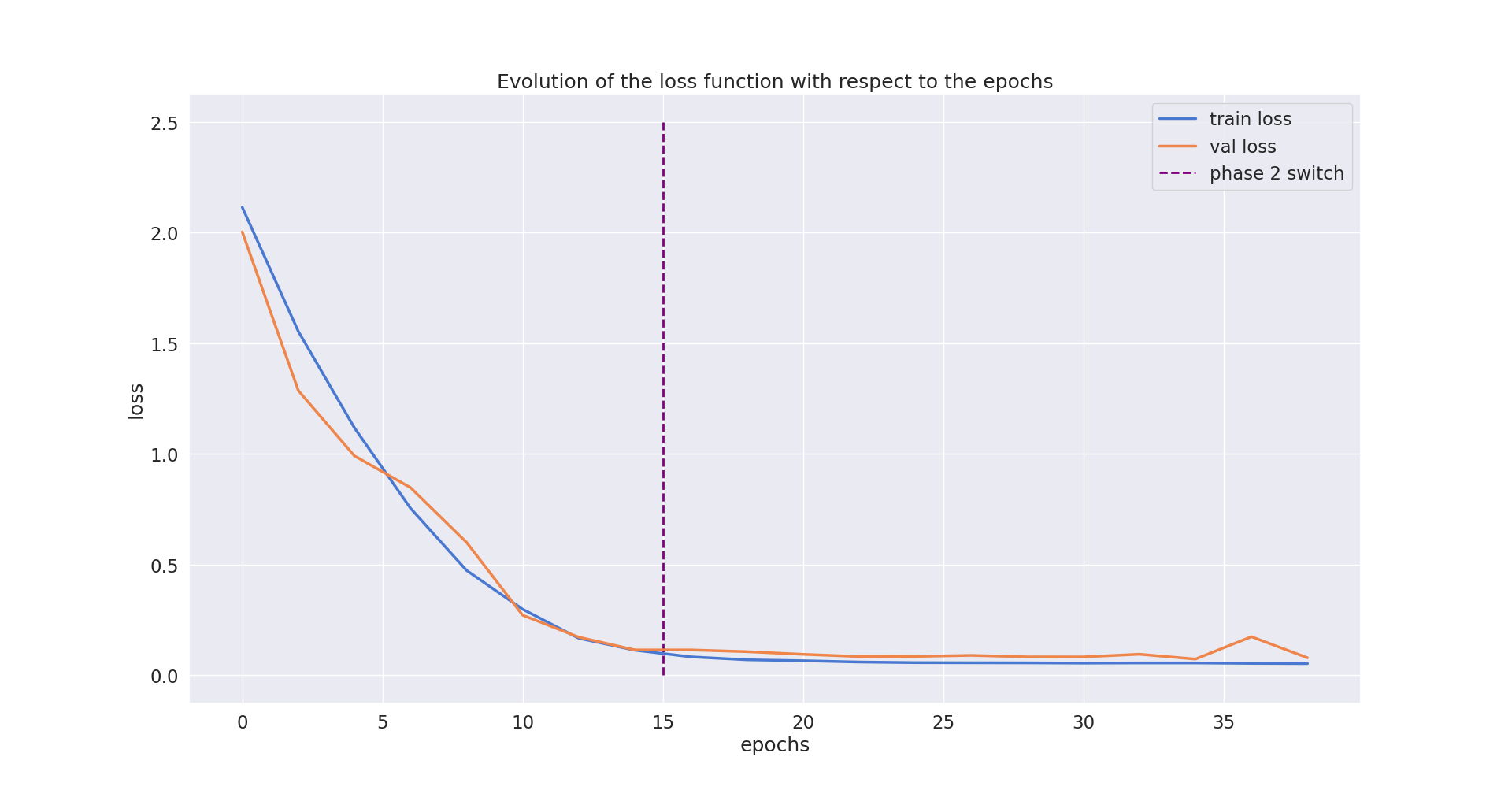

Supplement: S2 Fig — We display the evolution of both training and validation loss before and after the phase switch for the tumor classification task. (PNG) [file pcbi.1010921.s007.png]

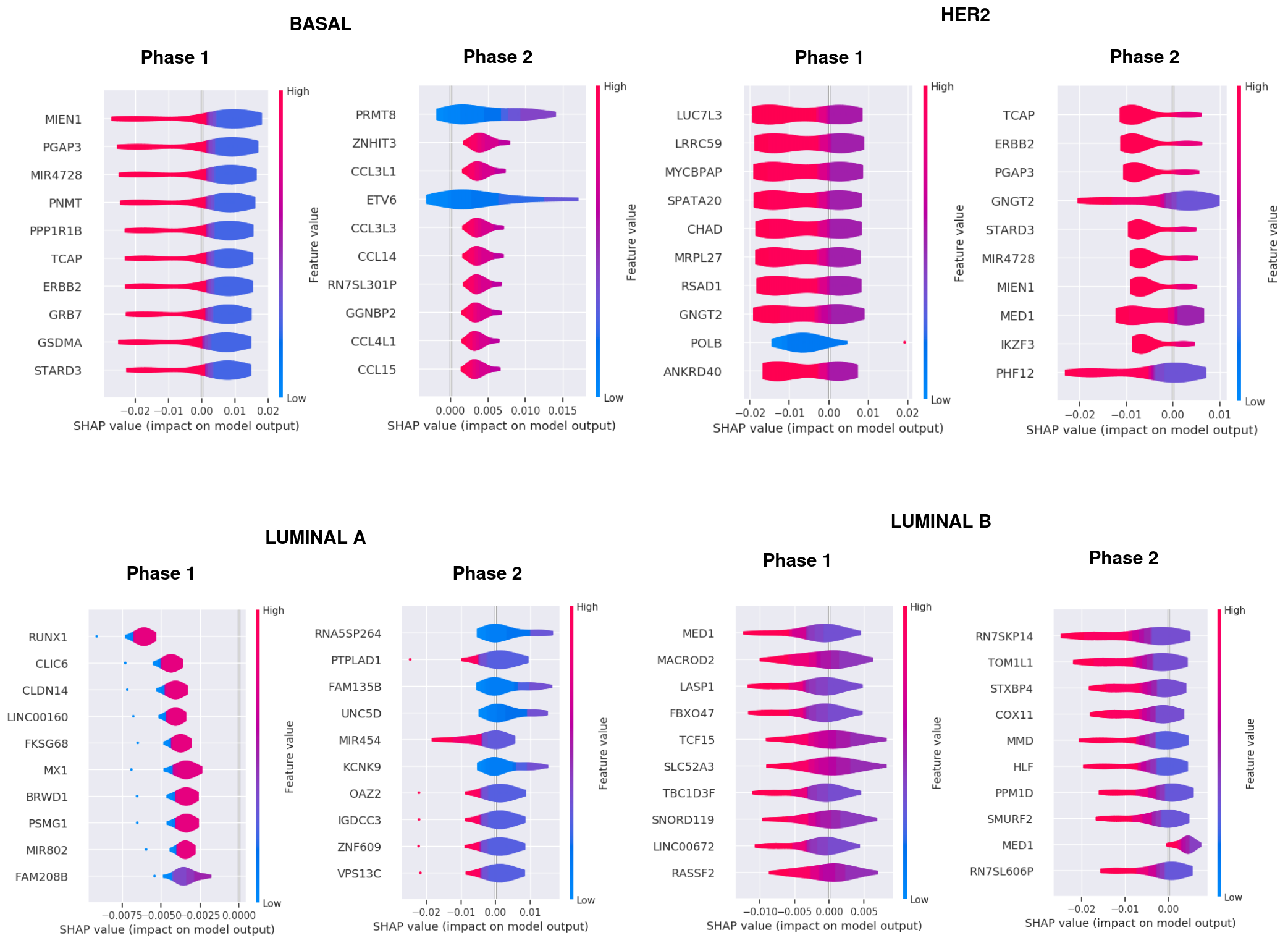

Supplement: S3 Fig — Computed SHAP values on the CNV data of the most relevant genes responsible for the discrimination between each subtype against the others using CustOmics for both integration phases. (PNG) [file pcbi.1010921.s008.png]

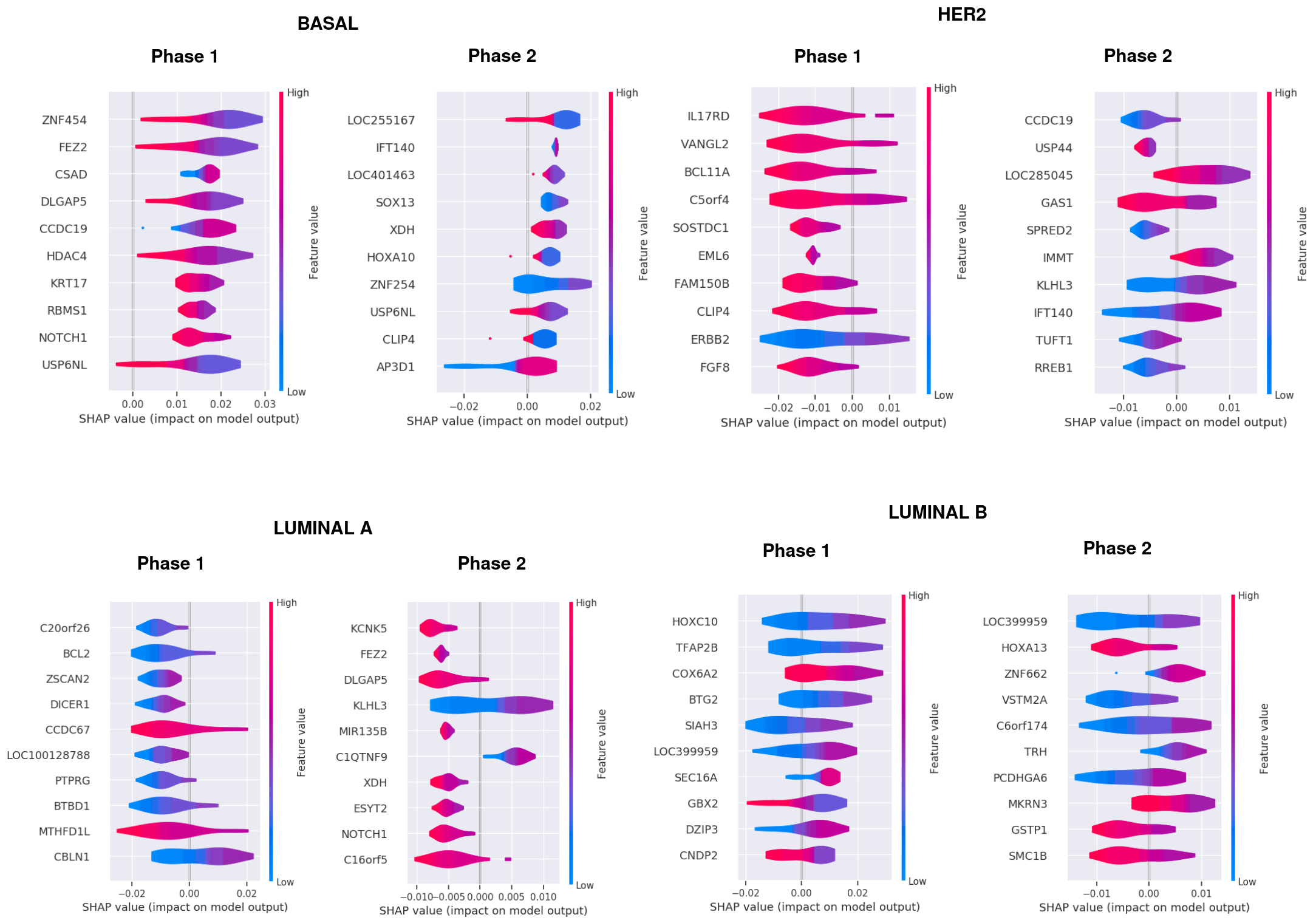

Supplement: S4 Fig — Computed SHAP values on the methylation data of the most relevant genes responsible for the discrimination between each subtype against the others using CustOmics for both integration phases. (PNG) [file pcbi.1010921.s009.png]
