# Supplementary material for: CustOmics: A versatile deep-learning based strategy for multi-omics integration
Source: PLoS Comput Biol. 2023 Mar 6;19(3):e1010921. doi: 10.1371/journal.pcbi.1010921 (PMC10019780; doi:10.1371/journal.pcbi.1010921)
Supplement: S1 Text — Explanations for SHAP Values and the computations behind. (PDF) [file pcbi.1010921.s010.pdf]

SHAP Values

February 12, 2023

SHAP values [LL17] help analyze which variables have an essential role in model predictions. Hence, it permits both a local and global interpretation of the model. For example, for binary classification, SHAP values let us know which variables have influenced classification as positive and which ones have influenced classification as negative.

Let $f : R^D \to R$ be the model to explain. We can use a simpler model $g : R^M \to R$ with $M \leq D$ that we will call the explaining model. Generally, explaining models use a simplified version x' of x such that there exists a transformation $h_x : R^M \to R^D$ where $h_x(x') = x$. Hence, g has to be an interpretable approximation of f, which means that for $z' \approx x'$, we want $g(z') \approx f(h_x(z'))$. One way to do this is to consider the additive features' attribution methods:

[Additive Features Attribution]

We call additive features attribution methods the set of interpretation methods having a linear explaining model with respect to binary features, i.e., an explaining model $g : R^M \mapsto R$ written as:

$$g(z') = \phi_0 + \sum_{j=1}^{M} \phi_j z'_j$$

where $z' \in \{0,1\}^M$, $z'_j = 1$ if z_j is present and $\phi_j \in R$ is the effect of the feature j.

Hence, the values ϕ will satisfy the following properties:

- **Local Precision**: For $x = h_x(x')$, we have $f(x) = g(x')$.
- **Lack of interest**: If simplified data represents the presence of features, non-present features do not have any effect: $x'_i = 0 \longrightarrow \phi_i = 0$.
- **Consistency**: If a feature's value increases or stays the same, so does its effect ϕ. Let $z'_{i=0}$ be the vector z' with the i-th entry null. Then for two models f and f' we have that if for all $z' \in \{0,1\}^M$:
$$f'(h_x(z')) - f'(h_x(z'_{i=0})) \geq f(h_x(z')) - f(h_x(z'_{i=0}))$$
Then
$$\phi_i(f', x) \geq \phi_i(f, x).$$

It has been proven in [LL17] that Shapley values are the only additive features attribution methods that satisfy those three properties. Hence, for a model f and an input vector $x \in R^D$, the i-th SHAP Value is given by:

$$\phi_i(f, x) = \sum_{z' \subseteq x'} \frac{|z'|!(M - |z'| - 1)!}{M!} [f(h_x(z')) - f(h_x(z'_{i=0}))] \quad (1)$$

where $|z'|$ is the number of non-zero values in z' and $z' \subseteq x'$ means all the vectors z' such that the non-zero values are a subset of the non-zero values of x'. We compute the model's prediction with and without a specific feature and witness the induced change. And we sum for all the combinations of possible features with the associated weight. SHAP Values are a direct consequence of this result, quantifying the importance of each variable for a complex predictive model. In fact, Shapley values defined by:

$$f(h_x(z')) = E[f(z)|z_S] \quad (2)$$

with S the set of non-zero features in z', are the only values that satisfy the previous requirements. Hence, SHAP Values can be interpreted as the difference between the observation's and mean predictions. However, the computation of SHAP Values is heavy (exponential complexity) for large datasets. This is why we can use specific SHAP models that consider the data structure to optimize the process (TreeShap for the case of Gradient Boosted Decision Trees).

References

[LL17] Scott M Lundberg and Su-In Lee. A unified approach to interpreting model predictions. In I. Guyon, U. V. Luxburg, S. Bengio, H. Wallach, R. Fergus, S. Vishwanathan, and R. Garnett, editors, *Advances in Neural Information Processing Systems 30*, pages 4765–4774. Curran Associates, Inc., 2017.
