# Supplementary material for: CustOmics: A versatile deep-learning based strategy for multi-omics integration
Source: PLoS Comput Biol. 2023 Mar 6;19(3):e1010921. doi: 10.1371/journal.pcbi.1010921 (PMC10019780; doi:10.1371/journal.pcbi.1010921)
Supplement: S2 Text — Definition of survival analysis and the notations used in the study. (PDF) [file pcbi.1010921.s011.pdf]

Survival Analysis

February 12, 2023

Survival outcome prediction is a regression task that models time-to-event distributions. The observations considered can either be uncensored if the event has been observed or (right-)censored if the event has not been observed before the last follow-up.

Notation:

- T^*, C are random variables corresponding respectively to the times of event and censoring.
- $T_i = min_{1 \leq i \leq n}(T_i^*, C_i)$ are the response variables actually observed. Here, we will only consider right censoring.
- $\delta \in \{0, 1\}^n$ an indicator vector such that $\delta_i = 1_{T_i^* \leq C_i}$ i.e. if $\delta_i = 0$ then the data is censored.
- For a given time t, we say that an individual i is at risk if its event time is located after t.

For T^* a real positive random variable representing the event time and $t \in R^+$, we denote by $f(t)$ its density and $F(t)$ its cumulative distribution function (CDF). We define the survival function as

$$S(t) = P[T^* > t] = 1 - F(t)$$

It corresponds to the probability that the event of interest occurs after time t. We also define the hazard function:

$$\mu(t) = lim_{dt \to 0} \frac{P[t \leq T^* < t + dt | T^* \geq t]}{dt}$$

It corresponds to the probability that the event occurs between time t and $t + dt$, knowing that the event hasn't occurred before t: it is also called the instantaneous death rate. We also consider the integrated hazard rate $H(t) = \int_0^t \mu(x)dx$
